# Surround yourself with creative people: How employee narcissism connects to creativity

**DOI:** 10.3389/fpsyg.2025.1646817

**Published:** 2025-09-17

**Authors:** Qiuyun Guo

**Affiliations:** School of Economics and Management, Taiyuan Institute of Technology, Taiyuan, China

**Keywords:** employee narcissism, creative coworkers, creative self-efficacy, creativity, social cognitive theory

## Abstract

Researchers have proposed that contextual factors in organizations can influence employee creativity and that this effect may vary as a function of employee personality. To extend the literature, this study proposed a theoretical model to explain the conditions under which narcissistic employees become more creative. This study theorizes that creative self-efficacy mediates the relationship between employee narcissism and creativity, and propose an important boundary condition—the presence of creative coworkers. Specifically, for narcissistic employees observing their coworkers engaging in creative behaviors increases their creative self-efficacy and subsequently increases their creativity. Our theoretical framework empirically tested through a study involving 247 full-time employees. The results indicate a positive relationship between employee narcissism and creative self-efficacy, which promotes creativity. Furthermore, the presence of creative coworkers moderates these relationships. This study clarifies inconsistent findings regarding the association between narcissism and creativity but also provide insight into the underlying mechanism based on social cognitive theory. This study offers managers insights into leveraging narcissistic employees—who are often considered more creative—by highlighting the critical role of creative coworkers. Managers should integrate narcissistic employees into innovation teams with highly creative peers to activate their creative potential through synergistic collaboration.

## Introduction

Employee creativity has long been recognized as crucial to organizational survival and the success of organizations (Anderson et al., 2014; Lin, 2007). It not only generates new and useful ideas but also enables their implementation (Amabile, 1988). Additionally, being the micro foundation of firm innovation (Liu et al., 2016), creativity can substantially contribute to the development of core competencies and distinct competitive advantage (Shalley et al., 2004). Given the escalating importance of employee creativity in dynamic contemporary environments, scholars and practitioners have extensively investigated various workplace antecedents. Employee personality has consistently emerged from empirical research as a crucial antecedent of creativity, encompassing traits such as proactive personality (Gong et al., 2010), creative self-efficacy (Mao et al., 2021; Tierney and Farmer, 2011; Zhao et al., 2023), and positive affect (Amabile et al., 2005; To et al., 2017). While studies have shed light on the influence of individual personality on creativity, delving into specific employee traits, such as narcissism, that directly impact creativity could significantly contribute to both theoretical advancement and practical application of employee creativity.

Narcissism is a stable personal trait, encompassing grandiosity, self-admiration, and an exaggerated perception of one's own skills and value (Campbell et al., 2011; Jonason et al., 2017; Wisse et al., 2015). Early studies primarily focused on leader narcissism and examined its effects (Braun, 2017; Hoffman et al., 2013; Owens et al., 2015). Subsequent research shifted attention to employee narcissism (Goncalo et al., 2010; Mao et al., 2021; Martinsen et al., 2019), revealing mixed results regarding its relationship with creativity. For example, a substantial corpus of empirical research has documented a positive correlation between narcissism and creativity (Ames et al., 2006; Furnham et al., 2013; Galang et al., 2016). Conversely, other investigations have suggested a more complex, double-edged sword effect of narcissism on creativity (Jonason et al., 2017), while Goncalo et al. (2010) argued that there is no significant correlation between narcissism and objective measures of creativity. Collectively, these findings suggest that the narcissism-creativity relationship is complex and likely moderated by contextual factors. Therefore, this study aims to reexamine this relationship and explore its underlying mechanisms.

According to social cognitive theory (Bandura, 1986), individuals are more likely to generate creative beliefs and engage in creative behaviors when they possess confidence in their ability to achieve desired outcomes. Consequently, narcissistic employees tend to bolster their creative self-efficacy because of their inflated self-perceptions and overconfidence (Mao et al., 2021). Creative self-efficacy acts as a mediator in the positive association between employee narcissism and creativity. Moreover, the social learning perspective (Shalley and Perry-Smith, 2001), which extends social cognitive theory, posits that individuals can enhance their creativity by observing and learning from creative role models. In an organizational setting, narcissistic employees may perceive their creative coworkers as potential rivals, viewing them as entities that motivate narcissistic employees to engage in learning activities and emulate creative behaviors. These coworkers serve as models by demonstrating creativity-related skills, employing effective strategies, and actively participating in creative endeavors (Bandura, 1986; Baron and Kenny, 1986; White and Mitchell, 1979). Therefore, the presence of creative coworkers can stimulate narcissistic employees to enhance their creative self-efficacy. Hence, the study also explores the moderating impact of creative coworkers.

This paper makes several theoretical contributions. First, it addresses the seemingly inconsistent creativity outcomes of employee narcissism and extends the understanding of the relationship between narcissism and creativity in modern organizations. The paper provides a framework for comprehending why and under what conditions narcissism can promote creativity. Second, based on social cognitive theory, the paper delves into the underlying mechanism explaining why narcissistic employees may exhibit creativity. By examining the mediating effect of creative self-efficacy, it uncovers psychological processes and provides a comprehensive understanding of the link between narcissism and creativity. Lastly, by adopting the learning perspective of the social cognitive theory, this paper explores the role of creative coworkers as a crucial boundary condition to understand the significant effects of role modeling on narcissists.

### Theory and hypotheses

#### Narcissism

Campbell et al. (2011) proposed that narcissism comprises three fundamental components: (a) a self-perception characterized by feelings of vanity and uniqueness, a sense of deserving special privileges, and strong aspiration for authority and recognition; (b) impaired interpersonal connections, including reduced empathy; and (c) self-regulatory strategies employed to maintain exaggerated self-perceptions. Scholars are interested in the conceptualization of narcissism and exploring its measurement. Grandiose narcissism and vulnerable narcissism are the two classic forms (Miller and Campbell, 2008). Both involve significant self-importance, entitlement, and arrogance; however, grandiose narcissism manifests as a highly agentic, dominant, and excitement-seeking personality, while vulnerable narcissism is associated with avoidance, high anxiety, and neuroticism. Considering our study's focus on how and why employee narcissism affects creativity and the greater scholarly emphasis on grandiose narcissism (Campbell et al., 2011; Reina et al., 2014), I limited our investigation to grandiose narcissism, which I evaluated using the Narcissistic Personality Inventory (NPI; Ames et al., 2006).

Studies of narcissism have focused on its outcomes from either an interpersonal or intrapersonal perspective. From an interpersonal perspective, narcissists are often hostile, exploitative, and unempathetic (Munro et al., 2005), resulting in low subordinate satisfaction (Bernerth, 2022), counterproductive work behaviors directed at supervisors (Braun et al., 2018), and employee organizational deviance (Zhang et al., 2018), despite the initial impression of narcissism being related to popularity (Back et al., 2010). In addition, the intrapersonal effects of narcissism have been a popular research topic and considered a “mixed blessing.” Narcissism is linked to unethical behaviors such as stealing, rule-breaking, and tax evasion (Watts et al., 2013), but it is also associated with corporate social responsibility (Petrenko et al., 2016) and indirectly promotes not only taking charge but also fostering radical and incremental creativity (Liu et al., 2022; Mao et al., 2021).

#### Creative self-efficacy

Creative self-efficacy refers to a collection of self-concepts that are essential for instilling individuals with the self-assurance to produce innovative outcomes (Tierney and Farmer, 2002). Unlike general self-efficacy, creative self-efficacy is specific to creativity and motivates individuals to identify and solve problems creatively. It is influenced by various contextual and personal factors. Such as contextual factors include perceived creative expectations from supervisors (Tierney and Farmer, 2011), transformational leadership (Afsar and Masood, 2017; Gong et al., 2009), task autonomy, high-quality leader-member exchange, and support for creativity (Mathisen, 2011; Park et al., 2021). Personal factors include creative role identity (Braun et al., 2018) and learning orientation (Tierney and Farmer, 2011). Research has consistently shown a positive correlation between creative self-efficacy and an individual's perception of their own competence (Afsar and Masood, 2017; Royston and Reiter-Palmon, 2019; Tierney and Farmer, 2011). In this study, I propose that narcissism, despite being a negative personality trait, may increase creative self-efficacy.

#### Employee narcissism and creative self-efficacy

Research has suggested that individual personality traits can significantly increase creative self-efficacy (Chong and Ma, 2010). Narcissistic individuals, characterized by overconfidence and inflated self-views (Hirschi and Jaensch, 2015), are likely to exhibit high levels of creative self-efficacy in work environments. First, narcissistic individuals possess a strong belief in their capability (Tierney and Farmer, 2002). They are assertive and inclined to exert effort to execute creative tasks effectively and produce innovative outcomes, which in turn help them maintain a positive self-image (Byrne and Worthy, 2013). Second, narcissistic employees possess robust psychological resources (Zhou et al., 2018), such as a desire to be positively perceived by others and a sense of entitlement. These resources cause them to have greater confidence in their work and greater efficacy in creative tasks than employees with fewer psychological resources. Third, narcissistic employees tend to exert control over their work for the sake of uniqueness and superiority, fostering self-affirmation of their creative capability (Cohen and Sherman, 2014). Based on these arguments, I hypothesize the following:

H1: Employee narcissism is positively related to creative self-efficacy.

#### Moderating effect of creative coworkers

Based on Bandura's (1986) social cognitive theory, Shalley and Perry-Smith (2001) developed a social learning perspective that suggests individuals can acquire relevant strategies and approaches for exhibiting higher creativity in their own work through the observation of role models' creative behavior. Creative coworkers who demonstrate and possess creative abilities are often regarded as exemplary figures in the workplace (Zhou, 2003). The information exchanged among such creative coworkers can significantly influence employees' self-assessments and personal development. Thus, I propose that creative coworkers can stimulate the cognitive processes of individuals with narcissistic tendencies, despite their typically inflated self-perception.

First, creative coworkers challenge the perception of uniqueness and superiority held by narcissistic employees, motivating them to participate in the learning process (Back et al., 2013). In a creative workplace environment where coworkers strive to generate new and valuable ideas and engage in change-oriented behaviors (Amabile, 1988, 1983), narcissistic employees tend to interact with and interpret the social cues of their creative colleagues. As a result of narcissistic employees' overconfidence and belief in their own abilities, the signals they receive from creative coworkers can enhance their learning behavior, which not only helps them maintain their sense of uniqueness and superiority but also enables them to receive recognition and respect from others.

Second, creative coworkers encourage narcissistic employees to engage in competitive behavior (Back et al., 2013). In a workplace setting, colleagues who exhibit creative behaviors and innovation tend to receive recognition from supervisors and are seen as role models (Zhou, 2003). Narcissistic individuals, perceiving themselves as capable and creative, may view these creative coworkers as threats to their previously enjoyed recognition, status, and admiration. To maintain their distinctiveness among coworkers, narcissists reinforce their belief in their own creative abilities, leading them to engage in more effective creative behaviors.

Third, the presence of creative coworkers encourages narcissists to adopt self-regulatory strategies (Campbell et al., 2011). Specifically, narcissists actively seek opportunities to attract attention and admiration and are inclined to showcase their achievements and overall life satisfaction (Sedikides et al., 2004). When narcissists interact with creative coworkers during collaborative work, they notice their coworkers' displays of creativity and draw comparisons with themselves (Campbell, 2000). To validate their creativity, narcissistic employees feel compelled to demonstrate their creative abilities to their coworkers and others.

In contrast, when narcissistic employees do not concern their creative coworkers, they are less likely to benefit from the stimulation of their social cognitive processes. Consequently, they are less inclined to exhibit their abilities and demonstrate creative self-efficacy. Therefore, I propose the following hypothesis:

H2: The presence of creative coworkers moderates the relationship between narcissism and creative self-efficacy, such that the relationship is positive and stronger when creative coworkers are present.

#### Creative self-efficacy and creativity

The agentic perspective with social cognitive theory (Bandura, 1986) proposes that individuals are more inclined to exhibit specific behaviors if they possess confidence in successfully accomplishing tasks. Thus, an individual's belief in their creative ability can influence their task approach, engagement in creative activities, and willingness to exert effort (Haase et al., 2018).

Creativity involves a process of generating and implementing novel ideas to question established standards (Goncalo et al., 2010). In this creative process, individuals require confidence, competence, and courage. As hypothesized above, narcissistic employees who exhibit high creative self-efficacy believe in their capacity to produce innovative outcomes and tend to overcome obstacles, solve problems, and approach tasks creatively. Accordingly, creative self-efficacy can trigger employees' intrinsic motivations, leading to a stronger belief that their work will be successful. Research has supported the positive relationship between creative self-efficacy and creativity (Liao et al., 2010; Wang et al., 2014), and a meta-analysis revealed the significance of this relationship (Haase et al., 2018). Thus, I propose the following hypotheses:

H3: Employee creative self-efficacy is positively associated with creativity.

H4: Employee self-efficacy mediates the positive relationship between narcissism and creativity.

Based on the aforementioned analysis, I propose an integrative model. Specifically, I expect narcissistic employees to exhibit a high level of creative self-efficacy in the presence of creative coworkers. Consequently, when confident in their ability to produce creative outcomes, narcissistic employees are likely to engage in creative activities. In contrast, when creative coworkers are absent, narcissistic employees may feel less compelled to increase their creative self-efficacy and participate in creative activities.

H5: The presence of creative coworkers moderates the indirect effect of narcissism on employee creativity via creative self-efficacy, such that the indirect effect is stronger in the presence of creative coworkers.

**Figure 1:**
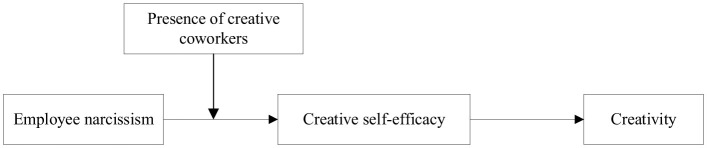
Research model.

The theoretical model is shown in Figure 1.

## Methods

### Participants and procedures

This study employed a multi-wave, time-lagged questionnaire survey to test the hypotheses. The participants were full-time employees from two information technology companies in western China. After obtaining permission from the executive managers of these companies, I collaborated with the human resources (HR) managers to facilitate the distribution of the questionnaires. To ensure the validity of our questionnaires, I conducted interviews with the HR managers, assuring them of the confidentiality of the results. Participants were selected from research and development divisions, which require considerable creativity. To incentivize participation, a reward of 10 yuan was offered to those who completed the initial questionnaire, and an additional 10 yuan was offered for completing the subsequent two questionnaires. The survey was conducted online using a reliable data-collection platform. Initially, I sent questionnaires to 350 employees. Identification codes were used to match leader and employee questionnaires across the three waves of data collection.

In wave 1, the participants provided demographic information and completed evaluations pertaining to narcissism and the presence of creative coworkers. Two weeks later, in wave 2, the participants completed evaluations regarding creative self-efficacy. Through data matching, I retained a sample of 275 employees, resulting in a response rate of 78.5%. In wave 3, two weeks after wave 2, I administered 275 questionnaires to a cohort of 45 direct supervisors of the aforementioned employees. These supervisors were requested to evaluate their subordinates' creativity, and I received 264 completed questionnaires. After excluding 17 incomplete or unsatisfactory questionnaires, I retained 247 complete and usable questionnaires, yielding a response rate of 89.8%. Male participants accounted for the majority (62.3%) of the sample which had an average age of 28.38 years. Additionally, a substantial proportion (73.3%) of the participants possessed at least a bachelor's degree.

### Measures

The survey items used in this study were initially developed in English. Following the recommended approach by Brislin (1980), I employed translation and back-translation to create Chinese versions of the survey items.

#### Narcissism

This study assessed employee narcissism by adopting the 16-item NPI developed by Ames et al. (2006). This scale uses a forced choice format, wherein participants are required to choose between a narcissistic response (“I like to be the center of attention”) and a non-narcissistic response (“I prefer to blend in with the crowd”). The narcissistic responses were coded as 1, while the non-narcissistic responses were coded as 0. Recent research demonstrated that compared with the Likert format, the original forced-choice format of the NPI exhibits similar construct validity and slightly greater reliability (Miller et al., 2018). The internal consistency of the narcissism measure, as measured by Cronbach's α, was 0.66.

#### Creative self-efficacy

Three items adopted from Tierney and Farmer (2002) were used to assess self-efficacy. An example item is “I am confident in my capacity to creatively solve problems.” Cronbach's α for creative self-efficacy was 0.73.

#### Presence of creative coworkers

The presence of creative coworkers was measured using a three-item scale developed by Zhou (2003). The participants were asked to indicate their level of agreement with three statements on a 5-point scale ranging from 1 (strongly disagree) to 5 (strongly agree). An example item is “I frequently observe my colleagues demonstrating creative behaviors in the workplace.” Cronbach's α for the presence of creative coworkers was 0.85.

#### Creativity

This study used the four-item scale developed by Farmer et al. (2003) to assess creativity. An example item is “This employee has the ability to generate innovative ideas”. The internal consistency of creative self-efficacy, as measured by Cronbach's α, was 0.73.

Control variables were included in accordance with creativity research (George and Zhou, 2007; Zhou, 2003). Demographic and other basic characteristics, including gender, age, education, and tenure, were controlled for. Gender was coded as a dichotomous variable (1 = women, 2 = men). Education was categorized into four levels: high school or below, college, undergraduate, and graduate education. Tenure was categorized into five levels: 1 = 1–5 years, 2 = 6–10 years, 3 = 11–15 years, 4 = 16–20 years, and 5 = more than 20 years.

## Results

Before performing hypothesis testing, I first conducted an exploratory factor analysis on measures of creative self-efficacy, presence of creative coworkers and creativity to examine their empirical distinctiveness. The analysis was performed using SPSS 26.0 software, employing an oblique promax rotation method. This approach helps produce a reliable factor structure by preventing factor collapse, as demonstrated by Browne (2001). The specific results of the rotated factor matrix for these three scales are presented in Table 1, which demonstrated satisfactory construct validity. All factors exhibited eigenvalues exceeding the threshold of 1.0, with all scale items loading significantly on their intended factors (loadings > 0.621) and maintaining acceptable discriminant validity (maximum cross-loading < 0.288).

**Table 1:** Items and factor analysis of creative self-efficacy, presence of creative coworkers and creativity.

**Items**	**Factor 1**	**Factor 2**	**Factor 3**
**Creative self-efficacy**			
I am confident in my capacity to creatively solve problems	**0.748**	0.074	0.286
I feel capable of generating novel ideas for difficult tasks	**0.793**	0.115	0.275
I consider myself skilled at generating novel ideas	**0.621**	0.297	0.198
**Presence of creative coworkers**			
I frequently observe my colleagues demonstrating creative behaviors in the workplace	0.094	**0.85**	0.146
My colleagues are innovative and creative	0.046	**0.864**	0.21
My colleagues actively seek new ideas for technologies, processes, tools, or products to improve work	0.267	**0.783**	0.24
**Creativity**			
This employee has the ability to generate innovative ideas	0.149	0.181	**0.742**
This employee seeks new ideas and ways to solve problems	0.288	0.118	**0.812**
This employee generates ground-breaking ideas related to the field	0.168	0.189	**0.829**
This employee is a good role model for creativity	0.181	0.174	**0.75**
Eigenvalue	4.558	1.507	1.116

All items were measured on a five-point scale (1 = strongly disagree to 5 = strongly agree). Bolded numbers indicate that the item has a high factor loading (> |0.6|) on its corresponding principal component. The loading value represents the correlation between the item and the principal component.

In addition, I conducted confirmatory factor analyses using Mplus 12.0 to ensure the discriminant validity of the narcissism (focal variable), creative self-efficacy, presence of creative coworkers, and creativity measures. The proposed four-factor model (i.e., narcissism, creative self-efficacy, the presence of creative coworkers, and creativity) exhibited satisfactory fit (χ^2^ [288] = 433.511, *p* < 0.01, CFI = 0.91, TLI = 0.90, root-mean-square error of approximation [RMSEA] = 0.05, standardized root-mean-square residual [SRMR] = 0.06). The fit statistics for the four-factor model were significantly better than those for a baseline one-factor model (χ^2^ [291] = 1,081.15, *p* < 0.01, CFI = 0.53, TLI = 0.49, RMSEA = 0.10, SRMR = 0.11). Thus, the CFAs indicated good discriminant validity in our measures.

Following the recommendations of Podsakoff et al. (2003), I used two approaches to test for common method variance resulting from the cross-sectional nature of our data. First, Harman's single-factor test was conducted by constraining the number of factors to one and employing the maximum likelihood method with varimax rotation. The first factor I tested accounted for 19.44% of the variance, suggesting the absence of one factor that could explain the majority of the covariance among the measures. This finding suggested that our data were not affected by common method variance. Second, an unmeasured latent method factor was added to the original four-factor (i.e., narcissism, the presence of creative coworkers, creative self-efficacy, and creativity) model to construct a five-factor model. When comparing the fit indices of the five-factor model with those of the four-factor model, I observed a slight increase in fit, but the change in comparative fit index (CFI) was only 0.003, which is less than the recommended threshold of 0.01 (Cheung and Rensvold, 2002). This result suggested that the two models were functionally equivalent, indicating no significant common method variance in our measures. Table 2 presents the means, standard deviations, correlations, and Cronbach's α values for all variables. Narcissism was significantly and positively correlated with creative self-efficacy (*r* = 0.20, *p* < 0.01) and creativity (*r* = 0.16, *p* < 0.01). Creative self-efficacy was significantly and positively correlated with creativity (*r* = 0.51, *p* < 0.01).

**Table 2:** Means, standard deviations, and correlations.

**No**.	**Variable**	***Mean***	***SD***	**1**	**2**	**3**	**4**	**5**	**6**	**7**	**8**
1	Gender	1.62	0.48								
2	Age	28.38	5.28	−0.01							
3	Tenure	1.39	0.74	−0.04	−0.01						
4	Education	2.73	0.82	−0.03	−0.004	−0.02					
5	Employee narcissism	0.31	0.17	−0.21^×^	−0.03	−0.07	0.02	(0.67)			
6	Creative self-efficacy	3.38	0.71	−0.01	0.1	−0.03	0.06	0.20^**^	(0.73)		
7	Presence of creative coworkers	3.41	0.84	−0.07	0.14	−0.02	−0.05	0.17^×^	0.40^**^	(0.85)	
8	Creativity	3.31	0.78	−0.08	0.23	0.024	−0.04	0.16^**^	0.51^**^	0.42^**^	(0.87)

** *p* < 0.01, Coefficient alpha is provided along the diagonal. “Mean” refers to the average value, and “SD” refers to the standard deviation.

### Hypothesis testing

Table 3 presents the results of the hierarchical regression analyses for creative self-efficacy (Models 1–4) and employee creativity (Models 5-10). Model 1 and Model 5 are the baseline models containing only the control variables. The subsequent models introduce independent variables, mediating variables, and interaction terms while retaining the control variables. Although the direct relationship between employee narcissism and creativity was not a focal hypothesis of this study, I first examined this prior relationship. After controlling for employee gender, age, tenure, and education, I found a significant relationship between employee narcissism and creativity (Model 6, β = 0.13, *p* = 0.009 < 0.05), similar to that (β = 0.18, *p* < 0.05) discovered by Wisse et al. (2015). Next, I used SPSS 26.0 to conduct hierarchical multiple regression analysis and test our hypotheses. Hypothesis 1 proposes a positive association between employee narcissism and creative self-efficacy, which is supported by the Model 2 findings in Table 3 (β = 0.15, *p* = 0.001 < 0.01).

**Table 3:** Results of hierarchical regression analyses.

**Variables**	**Creative self-efficacy**				**Employee creativity**					
	**Model 1**	**Model 2**	**Model 3**	**Model 4**	**Model 5**	**Model 6**	**Model 7**	**Model 8**	**Model 9**	**Model 10**
**Control variables**										
Gender	−0.01	0.05	0.07	0.08	−0.13	−0.07	−0.13	−0.11	−0.06	−0.1
Age	0.01	0.01	0.01	0.01	0.03	0.03	0.03	−0.02	0.02	0.02
Tenure	−0.03	−0.01	−0.01	−0.01	0.02	0.04	0.04	0.04	0.04	0.05
Education	0.05	0.05	0.06	0.06	−0.04	−0.04	−0.07	−0.07	−0.02	−0.06
**Independent variable**										
Employee narcissism		0.15^**^	0.11^*^	0.09^*^		0.13^**^		0.04	0.07	0.03
**Moderator**										
Presence of creative coworkers			0.27^***^	0.24^***^					0.30^***^	0.18^***^
**Interaction**										
Employee narcissism × Presence of creative coworkers				0.11^**^						0.04
**Mediator**										
Creative self–efficacy							0.39^***^	0.38^***^		0.32^***^
*R^2^*	0.01	0.05	0.19	0.22	0.06	0.09	0.31	0.32	0.23	0.36
*Adjusted R^2^*	0.001	0.04	0.18	0.2	0.04	0.07	0.29	0.29	0.21	0.34
*F*	0.93	2.97^**^	9.83^***^	9.84^***^	4.167	4.78^***^	21.93^***^	18.48^***^	11.87^***^	16.82^***^

*n* = 247.

* *p* < 0.05;

** *p* < 0.01;

*** *p* < 0.001 (two-tailed).

**Figure 2:**
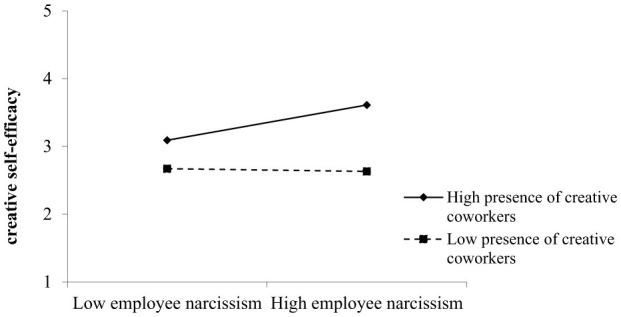
The moderating effect of the presence of creative coworkers.

Hypothesis 2 proposes that the presence of creative coworkers moderates the relationship between narcissism and creative self-efficacy. The results, presented in Table 3, show that the interaction between employee narcissism and the presence of creative coworkers was positively and significantly related to creative self-efficacy (Model 4, β = 0.11, *p* = 0.005 < 0.01). Figure 2 illustrates this interaction graphically. Simple slope tests revealed a significant positive relationship between employee narcissism and creative self-efficacy when the presence of creative coworkers was high (*B* = 0.16, SE = 0.04, *p* < 0.001), but a non-significant relationship when the presence of creative coworkers was low (*B* = −0.004, SE = 0.05, *p* = 0.93). This finding indicates that the presence of creative coworkers serves as a significant moderator in the relationship between narcissism and creative self-efficacy. Specifically, the positive effect of narcissism on creative self-efficacy is only significant when the presence of creative coworkers is high. Thus, it can be inferred that the presence of creative coworkers enhances the positive effect of employee narcissism on creative self-efficacy. These results support Hypothesis 2.

Hypothesis 3 proposes a correlation between employees' creative self-efficacy and creativity, which is substantiated by the Model 7 findings presented in Table 3 (β = 0.39, *p* = < 0.001). To test Hypothesis 4, I followed the procedure of Baron and Kenny (1986) to examine the mediating effect of creative self-efficacy. First, I observed a positive association between employee narcissism and creative self-efficacy. Second, Model 6 showed a significant and positive relationship between narcissism and creativity (β = 0.13, *p* = 0.001 < 0.05). Third, when employee narcissism and creative self-efficacy were concurrently included as predictors in the regression model (Model 7 in Table 3), the mediating factor of creative self-efficacy retained its significance in predicting creativity, and the relationship between narcissism and creativity became nonsignificant (β = 0.04, *p* = 0.298). These findings suggest a complete mediating effect of creative self-efficacy. Additionally, the results of a Monte Carlo simulation with 5,000 iterations support Hypothesis 4, showing an indirect effect of 0.08 between employee narcissism and creativity via creative self-efficacy, with a 95% confidence interval (CI) of [0.02, 0.14].

Hypothesis 5 contends that the presence of creative coworkers moderates the indirect effect of employee narcissism on creativity through employee creative self-efficacy. The PROCESS macro for SPSS, developed by Hayes (2013), was adopted to test for moderated mediation. As indicated in Table 4, when the presence of creative coworkers was low, the indirect effect of narcissism on creativity was not significant (*b* = −0.002; 95% CI [−0.08, 0.08]). However, this indirect effect was significant when the presence of creative coworkers was high (*b* = 0.09, 95% CI [0.04, 0.14]). The index of moderated mediation was also significant (*b* = 0.06; 95% CI [0.003, 0.11]), providing additional evidence of moderated mediation and thereby supporting Hypothesis 5.

**Table 4:** Indirect effects test of different conditions.

**Conditional indirect effect at high and low level of presence of creative coworkers**	**Effect**	***SE***	**Boot LLCI**	**Boot ULCI**
High presence of creative coworkers (+ SD)	0.09	0.03	0.04	0.14
Low presence of creative coworkers (−1 SD)	−0.002	0.04	−0.08	0.08
Index of moderated mediation	0.06	0.03	0.003	0.11

N = 247. 95% Confidence internal. Bootstrap sample size = 5000. Boot LLCI, bootstrap lower-limit confidence interval; Boot ULCI, bootstrap upper-limit confidence interval.

## Discussion

This study examined the influence of employee narcissism on creativity and explored the underlying mechanism linking these two factors. The research model was empirically supported by data gathered from a sample of 247 full-time employees. This study found that creative self-efficacy mediated the effect of narcissism on creativity and that the presence of creative coworkers moderated the relationship between employee narcissism and creative self-efficacy. The results of the data analysis provide invaluable insights and meaningful implications for both theoretical and practical advancements in the field for managers and researchers alike.

### Theoretical implications

First, this paper makes a valuable contribution to the literature on narcissism and creativity by examining the relationship between them. Our findings demonstrate a positive association between employee narcissism and creativity, extending current research in two important ways. Most studies of the antecedents of employee creativity have focused on individual personality traits, emphasizing positive attitudes and cognition (Zhou and Hoever, 2014). However, Wisse et al. (2015) suggested that individuals may benefit from possessing dark triad personality traits. Therefore, our results enrich the understanding of the antecedents of employee creativity by demonstrating the positive impact of employee narcissism on creativity. Our results also address the inconsistent findings of empirical studies by demonstrating that employee narcissism actually promotes creativity in modern Chinese organizations. This result is consistent with that of the empirical study conducted by Zhao et al. (2023). Additionally, our findings support the idea that narcissistic employees are motivated to learn from creative coworkers and that this positively influences narcissistic employees' own creativity. This finding sheds new light on the relationship between narcissism and creativity, both clarifying the association and elucidating the underlying mechanisms based on the principles of social cognitive theory.

Second, from the learning perspective of social cognitive theory, this study identified the presence of creative coworkers as a significant moderator of the relationship between employee narcissism and creative self-efficacy. Studies have explored various boundary conditions influencing self-efficacy, such as narcissist humility (Owens et al., 2015), supervisor expectations (Mao et al., 2021), and collectivist culture (Grijalva and Newman, 2015). Building upon this research, I examined the significance of creative coworkers, an unexplored factor in the context of the narcissism–creativity relationship. Creative coworkers are perceived as role models (Zhou, 2003) and are capable of stimulating narcissists' learning processes and bolstering their confidence and ability to generate creative outcomes. Our findings advance our understanding of the impact of narcissism by introducing a novel explanatory mechanism: The presence of creative coworkers facilitates the self-enhancement and cognitive processes of narcissistic individuals, consequently increasing creativity. Additionally, the findings contributed to the research of creative coworkers in Chinese culture. A traditional Chinese aphorism, “*Those close to vermilion become red, and those near ink turn black*,” suggests that associating with high-caliber individuals fosters personal improvement and excellence. Consequently, some Chinese organizations often promote creative role models, with managers expecting these exemplars to inspire others to learn and engage in creative activities (Shalley and Perry-Smith, 2001). In this study, I examined the moderating effect of creative coworkers on the relationship between narcissism and creativity. The results indicate that the presence of creative coworkers stimulates narcissists' creative behaviors, which aligns with the notion that being surrounded by creative people can benefit individuals.

Third, this research makes a valuable contribution to the literature on creative self-efficacy by providing insights into its antecedents. Research has suggested that contextual factors (e.g., job tenure, job complexity, supervisor behavior, and leadership style) (Puente-Díaz, 2016) or individual motivations (e.g., self-efficacy achievement goal) (Haase et al., 2018) can influence individuals' perceptions of their creative self-efficacy. Our results reveal how a generally negative personality trait such as employee narcissism can lead to creative self-efficacy when creative coworkers are present. Thus, our research enriches the literature on creative self-efficacy by uncovering a significant yet neglected antecedent of creative self-efficacy within the organizational context. Moreover, our findings enhance the understanding of the determinants of creative self-efficacy by demonstrating the potential for negative personality traits to foster creative self-efficacy when interacting with positive stimuli.

### Managerial implications

This study has valuable implications for organizations. Given the prevalence of narcissism in modern workplaces, identifying strategies that encourage narcissistic employees to engage in creative activities is crucial. First, when individuals with narcissistic tendencies experience high creative self-efficacy, they are internally motivated to actively participate in creative pursuits. Therefore, organizations should prioritize promoting and sustaining employees' creative self-efficacy by cultivating a creative climate, providing learning opportunities, and organizing competitions. Such initiatives could increase employees' confidence and creative capabilities. Second, creative coworkers play a role in stimulating narcissistic individuals to develop creative self-efficacy and enhance their creativity. Organizations can encourage employees' motivation to engage in creative behaviors by establishing a reward mechanism that encourages and recognizes creative employee role models. Furthermore, organizations can cultivate a strong creative culture that elevates the creative awareness of narcissistic employees. Third, organizations should implement measures to regulate the behaviors of narcissistic employees. While narcissism is often associated with ostentatiousness, it can be effectively channeled when properly guided and regulated. Supervisors can effectively communicate with narcissistic employees, clearly conveying managerial expectations and corresponding responsibilities. Additionally, demonstrating that work is meaningful can stimulate the self-motivation of narcissistic employees to engage in their tasks. By implementing these strategies, organizations can effectively leverage the strengths of narcissistic individuals while mitigating the potentially negative impacts of such individuals, ultimately fostering a creative and productive work environment.

### Limitations and future research

This study has limitations that future research should address. First, the data were collected in China; thus, the generalizability of the findings may be limited. Future research should analyze diverse cultural contexts to reevaluate the effects of narcissism and potentially identify differences among cultures.

Second, I employed a cross-sectional design. Although I used a multi-wave approach with a two-week interval to collect data, these methods did not constitute a true longitudinal study. The cross-sectional nature of the study made establishing causal relationships challenging, despite the models' consistency with theoretical assumptions. Future research should incorporate a longer time interval and employ experimental designs to improve the validity of causal inference.

Third, our results provide preliminary evidence of the positive effects of creative coworkers on employee narcissism. However, I did not consider the role of other important contextual factors that can influence employee narcissism, such as job complexity and task identity (Hackman and Oldham, 1975), which affect the self-motivation of narcissistic employees. Research should investigate the interactive effects of narcissism with job complexity or task identity to broaden the understanding of the contextual constraints associated with narcissism.

Moreover, while this study examined the mediating role of creative self-efficacy in the association between narcissism and creativity, our partial mediation model did not fully account for the relationship, underscoring the need to explore additional mediators. Research should explore attitudes, such as comparative identity (Johnson and Chang, 2006), that may mediate the positive effects of narcissism on outcomes.

### Conclusion

This study, grounded in social cognitive theory, investigates the relationship between employee narcissism and creativity, with creative self-efficacy mediating this link in the presence of creative coworkers. Our findings indicate that exposure to creative coworkers increases narcissistic employees' creative self-efficacy, which in turn leads to greater creativity. This underscores the organizational imperative to nurture a collaborative environment that leverages diverse traits to stimulate innovation.

## Data Availability

The raw data supporting the conclusions of this article will be made available by the authors, without undue reservation.
